# Transcriptional Repression by a bZIP Protein Regulates *Dictyostelium* Prespore Differentiation

**DOI:** 10.1371/journal.pone.0029895

**Published:** 2012-01-09

**Authors:** Beatriz Nuñez-Corcuera, Joanna L. Birch, Yoko Yamada, Jeffrey G. Williams

**Affiliations:** College of Life Sciences, University of Dundee, Dundee, United Kingdom; Cardiff University, United Kingdom

## Abstract

In response to the signaling polyketide DIF-1 DimB directly activates transcription of the ecmB gene in pstB cells; a subset of the prestalk cells that are the precursors of the basal disc. We show that the promoter of pspA, a prespore-specific gene, also contains a DimB binding site. Mutation of this site causes ectopic expression in the prestalk region and ChIP analysis shows that DIF-1 induces binding of DimB to the pspA promoter. DIF-1 represses pspA gene expression in a suspension cell assay but this repression is abrogated in a dimB null strain. These results suggest a coupled control mechanism, whereby the same DIF-DimB signaling pathway that directly activates ecmB gene expression directly represses pspA gene expression.

## Introduction

Biological systems frequently employ coupled control mechanisms to effect on-off switching because they ensure a tightly coordinated regulation. In the *Dictyostelium* asexual life cycle a single cell type can differentiate as either a prestalk or a prespore cell. This switch is governed by a form of coupled control whereby the same extracellular signaling molecule, DIF-1 (hereafter termed DIF), both induces certain types of prestalk differentiation and represses prespore differentiation [1], [2]. DIF is a chlorinated hexaphenone, produced by the prespore cells [3], [4]. There are multiple prestalk cell types and the differentiation of two sub-types, pstO and pstB cells, is induced by DIF [5], [6].

DIF acts as a direct inducer of the transcription of the *ecmA* gene and the *ecmA* promoter contains a distal region that directs expression in pstO cells and a proximal region that directs expression in pstA cells [7]. The distal region contains binding sites for DimB, a bZIP protein [8]. DimB is required for DIF inducibility of *ecmA* and DimB accumulates in the nucleus and binds to the *ecmA* promoter when cells are treated with DIF [8], [9]. DIF also induces expression of the related *ecmB* gene in pstB cells, the immediate precursors of the lower cup and outer basal disc of the culminant. This induction too depends directly upon DimB [10].

While there is some understanding of the transcription factors mediating prestalk induction by DIF, the prespore repression pathway is relatively uncharacterized. DIF represses expression of the commonly used markers of prespore differentiation, *pspA* and the two co-regulated spore coat protein genes, *cotB* and *cotC*. For *pspA*, where it has been studied in most detail, repression occurs within an hour of DIF addition and is mediated at the transcriptional level [1]. There is genetic evidence that both DimB and DimA, another bZIP protein that is a dimerisation partner of DimB, are involved in repressing prespore expression in prestalk cells; in a null strain for DimB (a dimB- strain) cotB:lacZ is ectopically expressed in the pstO region, as is pspA:lacZ in a dimA- strain [9], [11]. However, in a dimB- mutant a pspA reporter is not expressed in the pstO region [8]. This inconsistency between reporter behaviours may be explained by the existence of distinct signaling pathways for these two classes of prespore marker; *cotC* transcription is dependent on PKA activity while *pspA* transcription is not [12] and expression of *cotC* is highly dependent upon the amoebozoan-specific transcription factor CudA while *pspA* expression is not [13].

While there is genetic evidence that DimB forms part of the DIF signaling pathway that represses prespre gene expression in prestalk cells we do not know whether this is due to a direct effect of DimB on the pspA promoter or whether DimB forms part of a transcriptional cascade that exerts an indirect effect, via another transcription factor. Relatively little is known about the transcription factors that regulate prespore expression. The best characterised prespore promoter, that of *cotC*, contains multiple binding sites for the zinc-finger transcription factor GBF, binding regions for CudA and an essential AT-rich region of unknown binding capacity [14], [15]. The transcription factors that regulate *pspA* expression have not been identified at all but its promoter has been mapped by deletion analysis [16]. Here we identify the proteins that bind to one of the essential regions defined in that study [16], show that one of them is DimB and present evidence that DimB acts as a direct repressor of pspA.

## Results

### Affinity chromatography with a *pspA* promoter region purifies DimB

**Figure 1:**
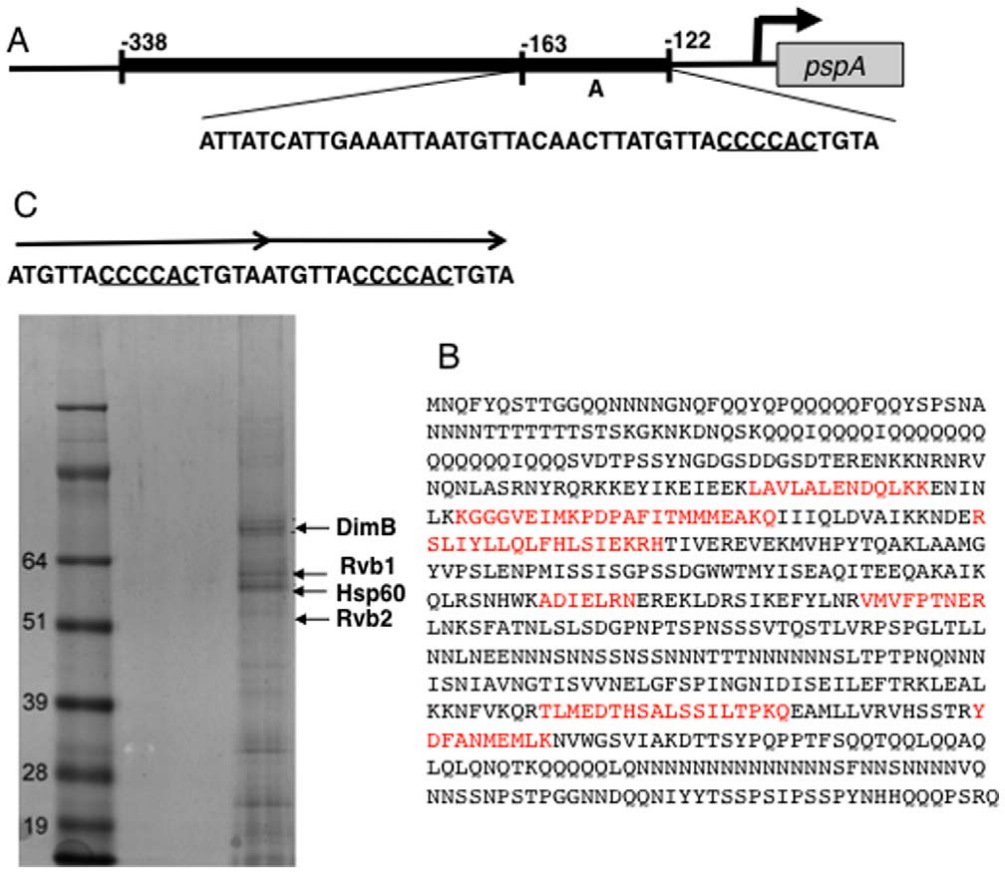
Identification of proteins that bind to the pspA promoter. A representation of the minimal promoter sequence required for *pspA* expression (thick line) showing the sequence of the region used in affinity chromatography, with a proposed DimB binding site underlined. (B) The combined peptide coverage for DimB in the two different purifications, described in Table 1. is shown in red. (C) Identification of proteins bound to a 16nt tandem dimer containing the proposed DimB site. Only those proteins with a deducible function are indicated and their scores in the mass spectrometry analysis are presented in Table 1.

When *pspA* promoter sequence downstream from −995 was subjected to 3′ to 5′ deletion, and fused to a *lacZ* reporter via the basal promoter elements of an actin gene, activity was retained to −122 but lost at −163 [1]. In order to identify transcription factors that interact with this region (region A in Fig. 1A) it was multimerised and used in affinity chromatography. Slug nuclear protein was bound to and eluted from the affinity resin twice and then subjected to gel electrophoresis. Those proteins with a score in mass spectrometry of >50 and where a likely function could be inferred from the protein sequence, are listed in Table 1. In two separate experiments one of the proteins bound by region A was identified as DimB (Fig. 1B).

**Table 1:** Mass spectrometry scores for selected of the proteins purified by affinity chromatography using region A.

Protein	Dictybase Gene ID and product	Score	
		A entire	A dimer
Rpb2	DDB_G0288257, polr2b, RNA polymerase II core subunit	298	
Rvb1	DDB_G0293226, RuVB-like protein 1	90	81
Rvb2	DDB_G0280775, RuVB-like protein 2		716
**DimB**	**DDB_G0291372, bZIP transcription factor**	**327**	**93**
HspC	DDB_G0272819, heat shock protein 32	131	
HspA	DDB_G0288181, heat shock protein 60		1014

Only known proteins identified as binding to region A (A entire), or the cap-site proximal tandem dimer (A dimer, Fig. 1B), and with a “Mowse” score of 50 or over are presented.

A cap-site proximal motif within region A, with sequence CCCCAC and which we term S, has a 5 out of 6 match to the sequence of R2; one of the two DimB binding sites within the *ecmA* promoter [8] and (Fig. 1A). DimB binding activity was mapped to that part of region A containing S by further DNA affinity chromatography. In order to facilitate annealing the proximal 16nt, containing site S, was synthesized as a dimer then multimerised (Fig. 1C). Affinity chromatography was repeated, using the multimer to generate the matrix, and DimB was again one of the proteins purified (Fig. 1C, Table 1). The dimer affinity chromatography also yielded two members of the INO80 chromatin re-modelling complex, Rvb1 and Rvb2 [17] and the heat shock protein Hsp60 but they were not analysed further.

### Gel retardation using region A maps two DimB binding sites

**Figure 2:**
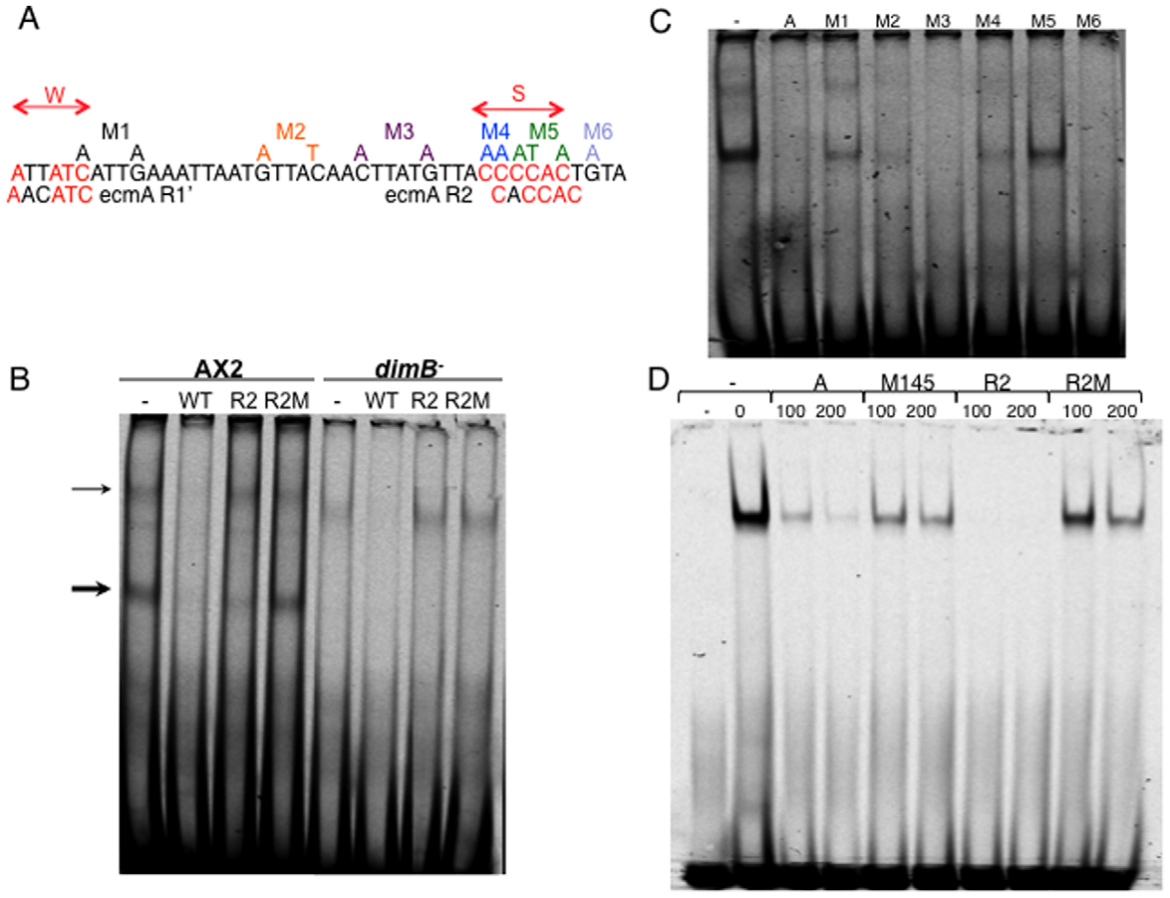
Mapping DimB binding sites in region A by gel retardation analysis. A) Alignment of regions S and W, the proposed DimB binding sites in region A of the *pspA* promoter, with the known DimB binding sites within the *ecmA* promoter: R2 and R1. Also indicated, above the sequence, are the positions of the point mutations used in scanning analysis of DimB binding. B) Total nuclear extracts obtained from Ax-2 and dimB- slug cells used in gel retardation with a region A probe. The competitors are the R2 and R2M sequences from within the *ecmA* promoter [8] C) Total nuclear extracts obtained from Ax-2 slug cells used in gel retardation with a region A probe. The competitors are region A itself and scanning mutants M1 to M6. D) Gel retardation with recombinant DimB using an A region probe. Competitors are: A itself, and oligonucleotide M145, containing region A with mutations M1, M4 and M5 that collectively mutate the S and W DimB binding sites. Again, the control competitors are the R2 and R2M sequences from within the *ecmA* promoter.

In order to map the DimB binding site in region A more precisely, nuclear extracts from parental Ax-2 and dimB- slug cells were used in gel retardation with pspA region A as probe (Fig. 2A). In the absence of competitors, and using an extract from parental Ax-2 cells (Fig. 2B), there is a major retarded band (thick arrow) and fainter, slower migrating complexes (thin arrows). Slower migrating material is also observed with a dimB- extract but the major retarded product is absent. This suggests that the major band is the DimB containing complex. This is supported by the competition behaviour observed with the R2 binding site from within the *ecmA* promoter [8] the major band is much reduced while the slower migrating material is unaffected (Fig. 2B). Oligo-nucleotide R2M contains point mutations that decrease competition for DimB binding to an *ecmA* probe [8] it is also ineffective as a competitor here, using region A as probe (Fig. 2B).

Region A contains, as stated above, a site S that has high sequence similarity to the R2 binding site (Fig. 2A). In a band-shift with region A as a probe, region A itself is a much more potent competitor than are M5 and M4, mutant forms of region A with S point mutated (Fig. 2A, 2C). These were generated as part of a mutation scanning of region A (Fig. 2A). As is usual with *Dictyostelium* promoter fragments, there is a very high proportion of A or T residues and so the scanning mutants were designed to remove the few GC containing clusters by replacement with A or T (Fig. 2A). Mutant M5 and the immediately adjacent mutant M4, which replaces the first two C residues in S, show reduced competition (Fig. 2B, 2C). So does mutant M1, located at the cap-site distal end of the sequence. The mutations in M1 have, however, a significantly weaker effect on competition than the mutations present in M5 (Fig. 2B, 2C). Hence it would seem that optimal binding to region A requires both S and W but that the contribution of W is lower. Consistent with this, we can recognize within W only a 4 out of 6 sequence identity to the invert complement form of R1: the weaker of the two DimB binding sites in the *ecmA* promoter (Fig. 2A) and [8].

We also analysed the binding of region A to DimB produced in *E. coli*. R2 and R2M show the expected behaviours in a band-shift using region A as a probe; R2 is a potent competitor while R2M is a much weaker competitor (Fig. 2D). In initial experiments, using mutant M1 or mutant M5 as competitors with a region A probe, neither mutation significantly reduced competition activity relative to the unmutated form (data not shown). Based on the above mutant scanning results with *Dictyostelium* extracts (Fig. 2C) we surmised that W might be playing a relatively more dominant role when using recombinant DimB. We therefore synthesized a multiply mutated form that targets both S and W. This mutant, M145, shows reduced competition activity relative to unmutated region A: supporting the notion of two sites with S playing the dominant role in vivo. We suppose that in *Dictyostelium* extracts modification of DimB, or interaction with other transcription factors, modulate its activity so as to decrease binding to W. This would make W and S mutually redundant under the artificial conditions of a band-shift assay using recombinant protein.

### Mutation of site S of pspA causes ectopic expression

**Figure 3:**
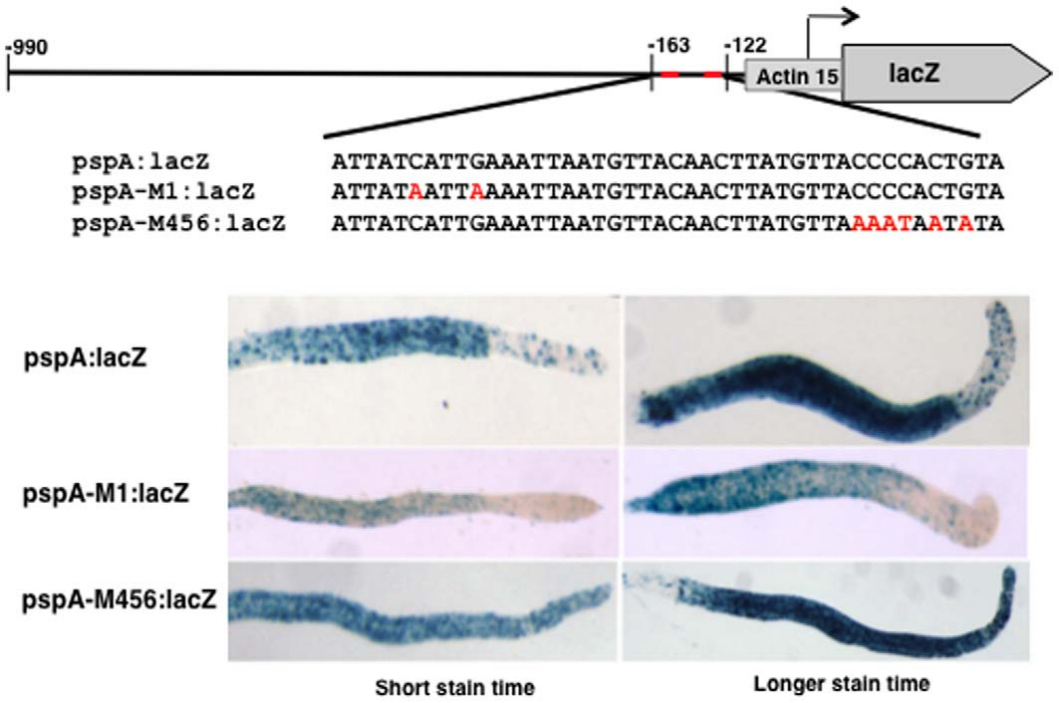
Expression patterns of *pspA* reporter fusions. The *pspA* promoter region −990 to −122 (pspA:lacZ), and versions of the same region containing a mutation of either the W (pspA-M1:lacZ) or S (pspA-M456:lacZ) sites. Expression patterns were established in standing slugs stained with X-gal.

We determined the effect of mutating S and W on *pspA* expression by creating *lacZ* reporter constructs (Fig. 3). The start point was pspA:lacZ, a *lacZ* promoter fusion construct with a distal end point at −990 and a proximal end point at −114 (numbered relative to the ATG initiation codon). In the S mutant construct, pspA-M456:lacZ, an 8nt region, containing S and spanning the positions of the three cap-site-proximal point mutants (M4, M5 and M6) analysed by band shift (Fig. 2A, 2B), was mutated to a random AT sequence. The W mutant construct, pspA-M1:lacZ, contains the two distal mutations present in M1. The three constructs were transformed into *Dictyostelium* cells, which were developed to the slug stage and stained for β-galactosidase.

The control, pspA:lacZ, gave the expected staining pattern, with strong staining throughout the prespore region and scattered staining cells in the prestalk region. A similar pattern was observed for the W mutant form, present in pspA-M1:lacZ. In contrast, the S mutant construct, pspA-M456:lacZ, showed staining in the prespore and the prestalk regions. This was true for both short and long times of staining, (Fig. 3). Thus mutation of the weaker DimB binding site, W, has no discernible effect on patterning but ablation of S causes ectopic expression in the prestalk region.

### DIF induces binding of DimB to the pspA promoter

**Figure 4:**
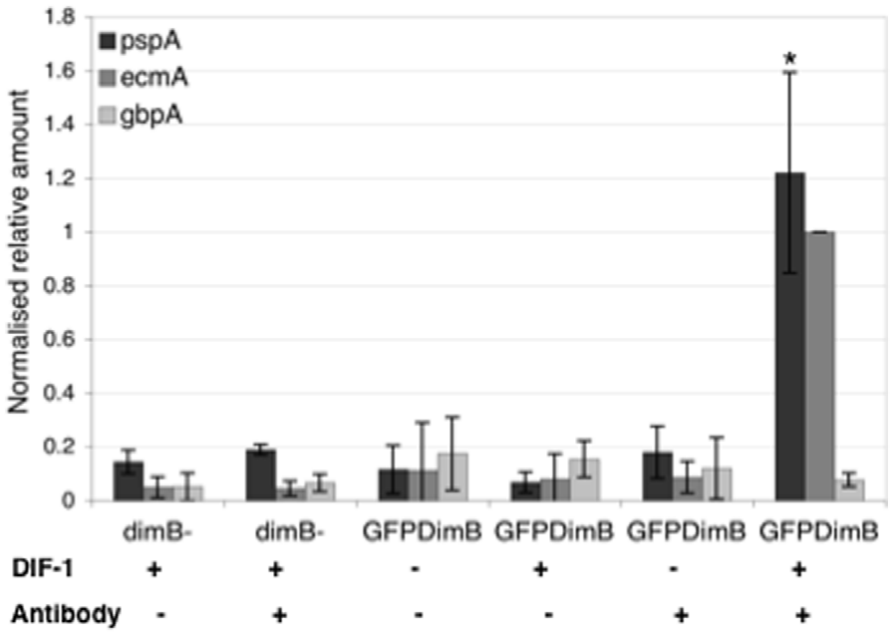
DimB binding to the pspA promoter *in vivo*. Cells were incubated with or without DIF and subjected to ChIP analysis. The absolute recoveries from the procedure varied from experiment to experiment, (three independent experiments with triplicate Q-PCR analyses in each). Therefore values are normalized to the induced signal for the *ecmA* positive control and are shown with their Standard Deviations. Student's paired T test was applied to the *pspA* analysis with and without DIF-1 and in samples immuno-precipitated from GFP-DimB transformant cells. As indicated by the asterisk the induction by DIF is significant with a P<0.05.

The possibility of a direct *in vivo* association of DimB with the *pspA* promoter was tested by ChIP analysis, using dimB- cells transformed with GFP-DimB, a fusion protein construct expressed from the *dimB* promoter. The presence of the GFP-DimB construct in the dimB- strain fully rescued the mutant phenotype (data not shown). Cells were mock induced or exposed to DIF for four hours and then subjected to ChIP analysis using the GFP tag for inmuno-purification. In the inmuno-precipitate, there is a DIF-dependent, antibody-dependent enrichment for *pspA* promoter DNA sequences, as assayed using Q-PCR (Fig. 4). In control dimB- cells, there is no such enrichment. Thus DIF induces binding of DimB to the *pspA* promoter.

### DimB is required for DIF-induced repression of pspA

**Figure 5:**
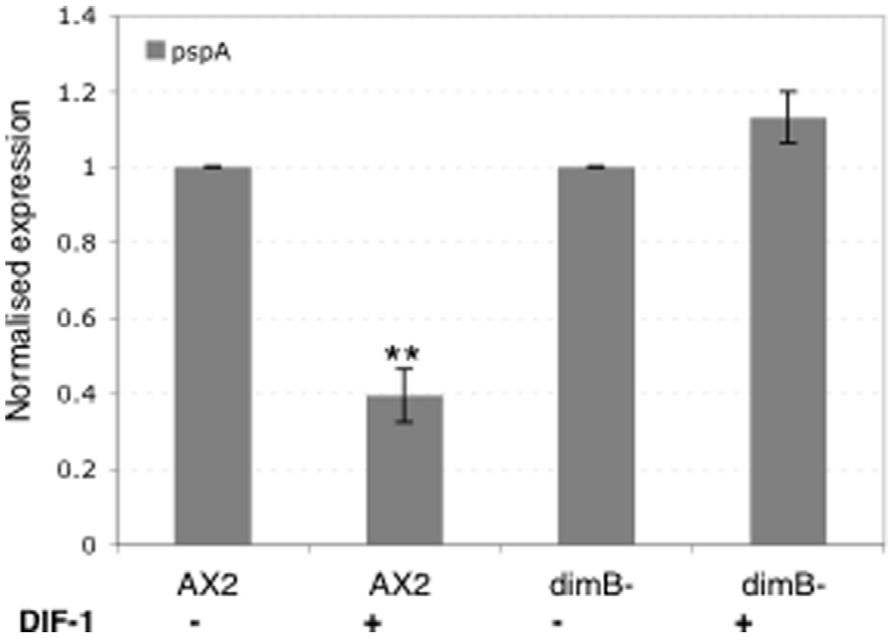
DIF repression of *pspA* expression. Disaggregated cells at the mound stage were incubated in the presence or absence of DIF-1. Q-PCR analysis of RNA samples was performed and the data is plotted as the mean of 3 independent biological repeats each performed in triplicate. The data is normalized to the expression level of *Ig7*, a constitutively expressed gene and that for each strain is normalized to the value without DIF. The mean results are shown with their standard deviations.

We observe an effect of the DimB null mutation on DIF response in suspension cells. When DIF is added to parental cells disaggregated at the mound stage it represses *pspA* mRNA accumulation but *pspA* expression is not repressed in dimB- cells (Fig. 5).

## Discussion

**Figure 6:**
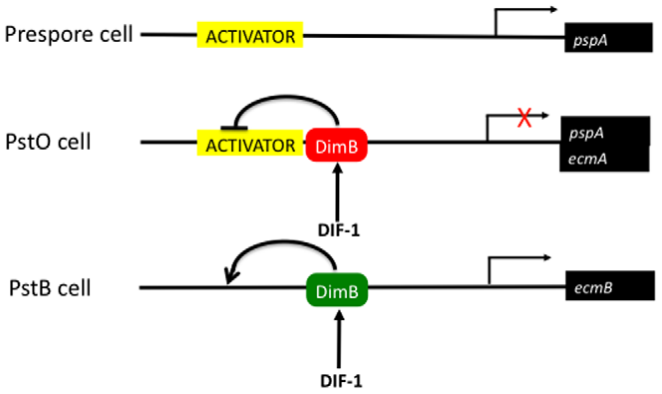
A scheme for the regulation of pspA gene expression. The scheme hypothesises a general activator of transcription (yellow-boxed) that has the potential to direct transcription in all cells in the slug. However, DimB acts in pstO cells in its repressor form (red-boxed) to prevent the activator functioning. Not shown here is a proposed functionally redundant repressor that can subsume the role of DimB as a repressor of pspA in a dimB- strain. The *ecmA* promoter is hyper-active in pstO cells of the DimB null strain, so is shown as being co-repressed by the DimB repressor form. In pstB cells the ecmB gene is directly induced by the activating form (green-boxed) of DimB.

These results suggest that activated DimB inhibits *pspA* expression in prestalk cells by directly interacting with the *pspA* promoter. Thus negative control confers the cell-type specific pattern of expression (Fig. 6). There is some prior evidence for negative control of prespore gene expression. PspC encodes a novel protein unrelated to PspA and deletion of a 160nt promoter segment causes ectopic expression in the pstO region of the slug [18]. One interesting difference between the two is that mutation of the strong DimB binding site in the pspA promoter leads to expression in pstO and pstA cells. This could be explained by an effect of the mutation on a separate transcription factor, that shares the DimB binding site but that represses pspA-specific expression in pstA cells.

Implicit in the above scheme is the existence of an activator that has the potential, in the absence of the repression mediated by DimB, to direct constitutive expression of *pspA*. Deletion of sequences located either downstream of −338 or upstream of −122 [16] eliminates expression; so the activators presumably bind multiple widely separated sites and may therefore be difficult to identify. The fact that the null mutant of DimB expresses a prespore construct, cotB:lacZ, in the pstO region supports this regulatory scheme [9]. However, there is no similar effect of the dimB null mutation on *pspA* expression [8]. This inconsistency leads us to propose a functional redundancy, manifest for pspA but not cotB, between DimB and one of the 18 other Dictyostelium bZIP proteins. Our observation that DIF-induced repression of pspA expression is abrogated in dimB- cells perhaps indicates that the unknown bZIP protein, which we propose to be functionally redundant with DimB, is not expressed or not activated in suspension cells. Interestingly, there is another apparent uncoupling between cotB and dimB here, because cotB remains DIF-repressible in a dimB- strain [9]. Again some kind of pathway-specific functional redundancy seems likely.

The repression exerted in anterior prestalk cells perhaps reflects a general property of DimB (Fig. 6); because, in a parental Ax2 but not in a parental AX4 background, the DimB null mutation leads to marked over-expression of the *ecmA* gene in pstO cells [8], [9]. DimB is not, however, a dedicated repressor; because *ecmB* expression in pstB cells is under direct, positive DIF-DimB regulation [10]. Thus, in the case of *pspA* and *ecmB* at least, DimB exerts coupled, “on-off” control (Fig. 6). It is possible that this control is simultaneously exerted in the same precursor cells, because during the early stages of slug formation DimB is nuclear-enriched in most or all cells and becomes nuclear enriched exclusively in pstB nuclei later [10]. Also implicit in all models of DIF-dependent patterning is the existence of some mechanism whereby the prespore cells, which are the source of DIF production [3], are themselves rendered insensitive to DIF-1.

The difference between the activator and repressor forms of DimB, hypothesised in Fig. 6, could be a simple result of the DimB nuclear concentration difference between pstO cells and pstB cells: low intranuclear DimB concentration in pstO cells favours repressor function while high intranuclear DimB favours activator function. Similar concentration dependent functional switching is well documented for a range of other transcription factors [19], [20], [21], [22].

## Materials and Methods

### Protein purification, mass spectrometry and gel retardation assay

*Dictyostelium* wild type strain Ax-2 (Gerisch isolate) and the dimB- strain were grown, and developed as previously described [8]. Total nuclear proteins were prepared from slug stage cells and used in affinity chromatography [8]. The samples were electrophoresed on an SDS gel and the excised bands analysed by MALDI-TOF mass spectrometry. Total Ax-2 and dimB- nuclear extracts, and His tagged DimB fusion protein synthesised in *E. coli*, were used in gel retardation assays [15].

### Fusion gene construction

The *pspA* promoter (region −990 to −114) was used to create mutations of the S and W binding sites within region A (Fig. 3). PCR products were cloned into Actin15ΔBam:gal [1] to provide basal transcription elements. GFP-dimB was created by amplifying 2.1 kb upstream of the *dimB* gene, then fusing the product upstream of GFP, which was in turn linked to the DimB coding region to give a translational fusion protein.

### ChIP analysis

GFP-DimB transformants, created in a dimB- background, and control dimB- cells were developed to the loose aggregate stage and mechanically disaggregated. The cells were induced by shaking at 4×10^6^ cells/ml in buffer, containing 2 mM cAMP and with or without 100 nM DIF-1 for 4 hours. After induction chromatin samples were analysed as in Zhukovskaya et al., 2006 except that immunoprecipitation was performed using GFP antibody (Roche Diagnostic, Germany) at 4°C overnight. QPCR was performed with immuno-precipitated DNA or control, total genomic DNA, using promoter-derived primers: *pspA*, forward CAAAAATAATATATTATGCTATGAATG and reverse CAGTGGGGTAACATAAGTTGTAAC (−321 to −223); *ecmA* forward TATTGCGTAATGGTTTTGCGGTC and reverse GGATTGTCGATCATATTTGATTAGTG (−453 to −417) and (as a control) *gbpA forward* CATATAACACGATTGTAAAAAAAAAC and reverse GTTTGTTTAAAATTGAGTGTGGGTTG (−731 to −583).

### DIF repression of gene expression

Disaggregated mound-stage cells were incubated at 4×10^6^ cells/ml for 4 hours with 2 mM cAMP and in the presence or absence of 100 nM DIF. RNA was extracted and analyzed by QPCR. All results were normalized to *Ig7*, a constitutively expressed gene. The *pspA* primers were: forward CGAATATACTACAAACCAATGT and reverse GTGGCAGTGATTTTACAAACTCCAC (+200 to +306).

## References

[1] Early AE, Williams JG (1988). A Dictyostelium prespore-specific gene is transcriptionally repressed by DIF in vitro.. Development.

[2] Kay RR, Jermyn KA (1983). A possible morphogen controlling differentiation in Dictyostelium.. Nature.

[3] Kay RR, Thompson CR (2001). Cross-induction of cell types in Dictyostelium: evidence that DIF-1 is made by prespore cells.. Development.

[4] Morris HR, Taylor GW, Masento MS, Jermyn KA, Kay RR (1987). Chemical structure of the morphogen differentiation inducing factor from Dictyostelium discoideum.. Nature.

[5] Saito T, Kato A, Kay RR (2008). DIF-1 induces the basal disc of the Dictyostelium fruiting body.. Dev Biol.

[6] Thompson CR, Kay RR (2000). The role of DIF-1 signaling in Dictyostelium development.. Mol Cell.

[7] Williams JG (2006). Transcriptional regulation of Dictyostelium pattern formation.. EMBO Rep.

[8] Zhukovskaya NV, Fukuzawa M, Yamada Y, Araki T, Williams JG (2006). The Dictyostelium bZIP transcription factor DimB regulates prestalk-specific gene expression.. Development.

[9] Huang E, Blagg SL, Keller T, Katoh M, Shaulsky G (2006). bZIP transcription factor interactions regulate DIF responses in Dictyostelium.. Development.

[10] Yamada Y, Nuñez-Corcuera B, Williams JG (2011). DIF-1 regulates Dictyostelium basal disc differentiation by inducing the nuclear accumulation of a bZIP transcription factor.. Dev Biol.

[11] Thompson CR, Fu Q, Buhay C, Kay RR, Shaulsky G (2004). A bZIP/bRLZ transcription factor required for DIF signaling in Dictyostelium.. Development.

[12] Hopper NA, Williams J (1994). A role for cAMP-dependent protein kinase in determining the stability of prespore cell differentiation in Dictyostelium.. Dev Biol.

[13] Fukuzawa M, Hopper N, Williams J (1997). cudA: a Dictyostelium gene with pleiotropic effects on cellular differentiation and slug behaviour.. Development.

[14] Powell-Coffman JA, Schnitzler GR, Firtel RA (1994). A GBF-binding site and a novel AT element define the minimal sequences sufficient to direct prespore-specific expression in Dictyostelium discoideum.. Mol Cell Biol.

[15] Yamada Y, Wang HY, Fukuzawa M, Barton GJ, Williams JG (2008). A new family of transcription factors.. Development.

[16] Early AE, Williams JG (1989). Identification of sequences regulating the transcription of a Dictyostelium gene selectively expressed in prespore cells.. Nucleic Acids Res.

[17] Shen X, Mizuguchi G, Hamiche A, Wu C (2000). A chromatin remodelling complex involved in transcription and DNA processing.. Nature.

[18] Hsu Y, Chang W, Newell PC, Gross JD (1999). A negative regulatory element in a prespore-specific promoter of Dictyostelium discoideum(1).. Biochim Biophys Acta.

[19] Kelley KM, Wang H, Ratnam M (2003). Dual regulation of ets-activated gene expression by SP1.. Gene.

[20] Sauer F, Jackle H (1993). Dimerization and the control of transcription by Kruppel.. Nature.

[21] Wallin JJ, Gackstetter ER, Koshland ME (1998). Dependence of BSAP repressor and activator functions on BSAP concentration.. Science.

[22] Kristjuhan A, Maimets T (1995). Protein p53 modulates transcription from a promoter containing its binding site in a concentration-dependent manner.. Eur J Biochem.

